# Effect of Straw Amendment on Soil Zn Availability and Ageing of Exogenous Water-Soluble Zn Applied to Calcareous Soil

**DOI:** 10.1371/journal.pone.0169776

**Published:** 2017-01-12

**Authors:** Yanlong Chen, Juan Cui, Xiaohong Tian, Aiqing Zhao, Meng Li, Shaoxia Wang, Xiushaung Li, Zhou Jia, Ke Liu

**Affiliations:** College of Natural Resources and Environment, Northwest A&F University, Key Lab of Plant Nutrition and the Agri-environment in Northwest China, Ministry of Agriculture, Yangling, Shaanxi, China; Institute of Materials Science, GERMANY

## Abstract

Organic matter plays a key role in availability and transformation of soil Zn (zinc), which greatly controls Zn concentrations in cereal grains and human Zn nutrition level. Accordingly, soils homogenized with the wheat straw (0, 12 g straw kg^-1^) and Zn fertilizer (0, 7 mg Zn kg^-1^) were buried and incubated in the field over 210 days to explore the response of soil Zn availability and the ageing of exogenous Zn to straw addition. Results indicated that adding straw alone scarcely affected soil DTPA-Zn concentration and Zn fractions because of the low Zn concentration of wheat straw and the high soil pH, and large clay and calcium carbonate contents. However, adding exogenous Zn plus straw increased the DTPA-Zn abundance by about 5-fold and had the similar results to adding exogenous Zn alone, corresponding to the increased Zn fraction loosely bounded to organic matter, which had a more dominant presence in Zn reaction than soil other constituents such as carbonate and minerals in calcareous soil. The higher relative amount of ineffective Zn (~50%) after water soluble Zn addition also occurred, and at the days of 120–165 and 180–210when the natural temperature and rainfall changed mildly, the ageing process of exogenous Zn over time was well evaluated by the diffusion equation, respectively. Consequently, combining crop residues with exogenous water soluble Zn application is promising strategy to maximize the availability of Zn in calcareous soil, but the higher ageing rate of Zn caused by the higher Zn mobility should be considered.

## Introduction

Recent estimates have suggested that ~50% of the global population is at risk of Zn deficiency. Zn deficiency is a major public health problem in many countries, especially in regions where people rely on cereal–based food with small Zn concentrations [1]. Several studies have shown that the low availability of Zn in soil rather than the small amount of total Zn is the main reason for the widespread Zn deficiency in cereal crops [2]. Zn fertilizer application is an effective practice that is used globally to improve Zn availability in soil [3, 4]. However, research has shown that the effects of Zn fertilizer on plant Zn concentrations are limited in calcareous soil [5]. Such limited effects might be mainly attributed to the poor diffusion of free Zn to plant roots, which was minimized by the variety of physical and chemical constraints (e.g., high pH and large carbonate and clay contents) [6]. Water-soluble Zn fertilizer becomes slowly but steadily more stable and immobile over time [7], a process known as ageing, which leads to a decrease in Zn solubility, mobilization and consequently Zn fertilizer efficiency.

Organic matter plays a key role in governing the availability of soil Zn [8]. Exogenous organic materials not only release free Zn into soil solutions by decomposition, but also change the original solubility and mobilization of soil Zn by the formation of Zn organic complexes [9]. Organic amendments were shown to alter the distribution of Zn precipitated by calcium carbonate, which is supposed to be a major factor in the loss of Zn availability in calcareous soil [7]. In addition, the adsorption and fixation of Zn takes place by the slow diffusion of available Zn into iron (Fe) and manganese (Mn) oxides and clay minerals, and this is also closely related to the change in the amount of Zn bound to organic matter [10]. The effect of organic matter on the availability of soil-Zn depends on the maturity of organic amendments. The availability of Zn is small where mature organic materials are present such as compost because of the formation of stable organic complexes with organic matter such as humic acid [9]. In contrast, rapidly degradable organic matter added to soil effectively dissolves originally insoluble Zn, which improves its solubility and availability in soil-plant systems because of water-soluble or labile organic compounds rich in functional groups (e.g. amino, carboxyl, and phenolic) that have strong chelating abilities [11]. For example, Aghili et al. [12] found green manure of red clover and sunflower amendments to calcareous soil raised grain Zn concentration in bread wheat with the increased DTPA-extractable Zn in soils. Habiby et al. [13] considered that the Zn concentration of wheat grain increased owing to the increase of soil dissolved organic matter after the incorporation of plant residues such as safflower and clover. Even, Sinha and Prasad [14] found that the direct addition of mobile organic chelating agents(e.g., fulvic and citric acids) enhances the rate of Zn diffusion into roots and consequently Zn uptake of wheat plants in calcareous soil. Similar results revealed that labile organic matter excreted by roots improved the diffusion of Zn from soil to roots and consequently maximized Zn uptake during the crop growing season [15].

In addition to manure and compost, the incorporation of crop straw into arable soil is currently strongly encouraged and widely used as an acceptable and environmentally friendly organic amendment all over the world. However, there is little information on the effects of crop straw amendments on the activity and fractions of Zn and the diffusion and ageing of exogenous Zn in calcareous soil. Therefore, here we hypothesize that (i) straw application effectively increases the Zn availability in arable soil through increasing the amount of Zn bound to soluble organic matter and reducing the accumulation of Zn in less readily available fractions such as Zn associated with calcium carbonate, Fe and Mn oxides, and clay minerals and (ii) straw incorporation has the potential to increase Zn diffusion and retard ageing of exogenous soluble fertilizer-Zn. The objective of this research was to evaluate the effect of straw amendment on the plant available soil-Zn concentration by determining the diethylene triaminepentacetate acid (DTPA) extractable Zn and Zn speciation, and on the ageing rate of the soluble fertilizer-Zn by the diffusion model in calcareous soil.

## Materials and Methods

### Experimental site

The experiment was conducted from November 2010 to June 2011at the Experimental Farm (525 m above sea level, 34°17′N, 108°04′E)of Northwest Agriculture and Forestry University, Yangling, Shaanxi Province, China. The climate is semi–humid and prone to drought with an annual average temperature of 13°C and an annual average rainfall of 632 mm. The soil is classified as an Earth-cumulic Orthic Anthrosol in the Chinese soil taxonomy, an Anthrosol in the FAO soil taxonomy, and an Inceptisol in the USDA soil taxonomy [16–18]. The main properties of the topsoil (0–20 cm) are as follow: pH 7.98 ± 0.12, 7.83 ± 0.23 g organic carbon kg^-1^, 63.5 ± 10.1 g carbonate kg^-1^, 69.8 ± 5.20 mg total-Zn kg^-1^, 271 ± 52.4 g sand kg^-1^ (0.02–2 mm), 408 ± 59.2 g silt kg^-1^ (0.002–0.02 mm) and 321 ± 20.6 g clay kg^-1^ (<0.002 mm). The DTPA-Zn concentration is 0.76 ± 0.08 mg Zn kg^-1^, which exceeds the critical deficiency level for DTPA extractable Zn in soil of 0.5 mg Zn kg^-1^ [1]. Lu et al. [5] indicated that soil is potentially Zn deficient when concentrations are 0.5–1.0 mg DTPA-Zn kg^-1^.

### Experimental procedure

A completely randomized design incubation experiment was conducted in the field of winter wheat (*Triticum aestivum* L.). The treatments included (i) no Zn and no straw added (control, CK), (ii) straw addition alone (Straw), (iii) soluble Zn addition alone (Zn), and (iv) straw plus soluble Zn additions (Straw+Zn). The wheat straw amendment was 12 g kg^-1^ dry soil (equal to 27 Mg ha^-1^ with 1.5-fold of the conventional application rate). The straw’s Zn concentration was 6.7 mg Zn kg^-1^, and its C:N ratio was 82:1. Zn sulphate as a water soluble fertilizer-Zn (with 21% Zn) was used at 7 mg Zn kg^-1^ dry soil (equivalent to the conventional application rate of 15 kg Zn ha^-1^).

Prior to the start of the experiment, topsoil (0–20 cm) was collected from the field and wheat straw from the previous season. Both soil and straw were air-dried, ground and passed through a 2-mm sieve. Small portions of these samples were used for determining the basic chemical properties of the soil and straw, and the remaining portions were stored for future use. On 15 October 2010, following the application of urea (with 46% N) at 120 kg N ha^-1^ and calcium superphosphate (with 8% P) at 44 kg P ha^−1^, winter wheat was sown at a row distance of 30 cm. On 15 November 2010, 4.2 kg of the sieved dry soil was mixed thoroughly with the prepared straw or/and fertilizer-Zn according to treatments. Meanwhile, urea was added to adjust the C:N ratio to less than 25:1 for all treatments and then the soil moisture was adjusted to 20% by deionized water. The mixtures of each treatment were divided into 21 equal portions and put individually into nylon mesh bag (10 cm×15 cm, 0.15-mm pore diameter). Next, a plot of 10 m×6 m (60 m^2^) was selected from the wheat field with 20 rows of wheat and 84 bags (labelled according to the treatment) were buried randomly between the wheat rows (1.5-m interval, 4–5 bags per row and at 25-cm depth). After 60, 120, 150, 165, 180, 195 and 210 days, three bags (replicates) of each treatment were removed randomly and the soil samples were collected. Small portions as fresh sample were stored at 4°C and the remaining portions were air-dried, sequentially passed through 1-and 0.25-mm sieves, and stored at 25°C for future use. During the entire experimental period, no water was supplied by irrigation in the field and the other management (e.g. pesticide and herbicide) was conducted manually according to the standard field practices for this region [5].

### Sample measurement

The sieved straw was incinerated at 525°C for 6 h, and then dissolved with 50% (v/v) nitric acid for the analysis of straw Zn concentration [5]. Straw nitrogen was analyzed by Kjeldahl digestion, and organic carbon of soil and straw by the Walkley-Black method [19]. Soil carbonate was determined by measuring the production of CO_2_ following dissolution with hydrochloric (HCl) acid [20]. Soil particle size distribution was determined by the dry sieving, as described by Lyles et al. [21]. Soil pH was measured with a pH meter (0.01 M CaCl_2_ at a soi:water ratio of 1:2.5). KMnO_4_ oxidizable carbon for fresh soil sample was defined as labile soil organic carbon (LOC) and measured using the method described by Blair et al. [22].

Soil available Zn concentration (DTPA-Zn) was extracted with 0.005MDTPA in 0.01M CaCl_2_ buffered at pH 7.3 with 0.1M triethanolamine (TEA) at a soil:water ratio of 1:2. Soil Zn fractionation was carried out with a modified Tessier's sequential extraction procedure as described by Lu et al. [5]. Each sample was placed into a 50 ml centrifuge tube and the operationally defined Zn fractions were obtained as following: The exchangeable and water-soluble (EXCH) fraction was extracted with 1 M Mg(NO_3_)_2_ at pH 7 for 2 h. The Zn in organic matter fraction with low bonding forces (LOM) was extracted with 0.1 M Na_4_P_2_O_7_ (pH 9.5) for 2 h. The Zn fraction in carbonate (CARB) was extracted with 1 M NaOAc-HOAc at pH 5 for 5 h. The Zn fraction in manganese oxide (MNO) was extracted with 0.1 M NH_2_OH·HCl at pH 7 for 30 min. The Zn in organic matter fraction with high bonding forces (HOM) was extracted with 1 M Mg(NO_3_)_2_ at pH 7 for 2 h after digesting the soil sample twice with 5 ml of 30% H_2_O_2_ (pH 2) at 80°C water bath for 2 h. All Zn fractions were extracted at a solution to soil ratio of 10:1 on a dry weight basis and shaken at 25°C. Residual Zn (RES) was calculated by subtracting the sum of above five fractions from total Zn that was digested with HCl-HNO_3_-HClO_4_-HF. All the resulting solutions for Zn concentrations of straw and soil were analyzed by an atomic absorption spectroscopy (Z-2000, Shanghai, China).

### Calculation of ageing rate

With time, the Zn added into soil continually and slowly decreases in activity and extractability, and changes to more stable forms [4]. Numerous researches attribute this to the slow diffusion of Zn into soil particles with concurrent adsorption onto oxide and clay mineral surfaces and precipitation of Zn carbonates [7]. If this diffusion is considered to be radial assuming that the aggregates are spherical, the diffusion equation for a constant diffusion coefficient takes the form [23]:

$$\frac{\partial C}{\partial t}=D\left(\frac{\partial^{2}C}{\partial t^{2}}+\frac{2}{a}\frac{\partial C}{\partial a}\right),$$ (1)

where *C* is the concentration of diffusing Zn, *D* is the diffusion coefficient, *a* is the radius of the spherical particle, and *t* is time. When the diffusing Zn concentration is initially uniform (*t* = 0), the diffusion equation can be described according to the research of Ma and Uren [24]:

$$\frac{X}{X_{m}}=6\sqrt{\frac{Dt}{\pi a^{2}}}+A=6\sqrt{\frac{D_{a}t}{\pi }}+A,$$ (2)

where *X* is the concentration of unavailable Zn (DTPA-unextractable-Zn)at time *t*, *X*_*m*_ is the initial concentration of the diffusing Zn, *D*_*a*_ is the apparent diffusion coefficient and equal to *D/a*^*2*^, and *A*is a constant which represents the initial sorption of Zn in soil.

The ratio *X/X*_*m*_ was defined as the relative amount of ineffective Zn (*Zn*_*RI*_) by Ma and Uren [4]. It is calculated from:

$$Zn_{RI}=\frac{X}{X_{m}}=\frac{Zn_{fert}+Zn_{soil}-Zn_{t}}{Zn_{fert}+Zn_{soil}}$$ (3)

where *Zn*_*fert*_ is the concentration of fertilizer-Zn added to the soil and *Zn*_*soil*_ is the endogenously initial DTPA-Zn concentration in soil (*t* = 0). The sum of *Zn*_*fert*_ and *Zn*_*soil*_ is equivalent to *X*_*m*_. *Zn*_*t*_ is the DTPA-Zn concentration in the Zn or Straw+Zn treatment at the *t* time. Combining Eqs (1) and (2) gives:

ZnRI=Znfert+Znsoil−ZntZnfert+Znsoil=6Dtaπ+A (4)

Ma and Uren [4] reported that plots of *Zn*_*RI*_ versus the square root of time was approximately linear for *Zn*_*RI*_≈0.5, consistent with Eq (4). Accordingly, they obtained for the ageing rate of added Zn at any particular time:

$$Ageing\;rate=\frac{dZn_{RI}}{dt}=\frac{d\left(6\sqrt{\frac{D_{a}t}{\pi }}+A\right)}{dt}\;=\;3\sqrt{\frac{D_{a}}{\pi t}}$$ (5)

### Statistical analyses

The experimental data were analyzed statistically with SAS software version 9 (SAS Institute, Cary, NC, USA). Two-way analysis of variance (ANOVAs) were used to investigate the effects of straw and zinc application and their interaction on DTPA-Zn concentrations, soil pH, and LOC concentration at the given incubation days. The variations in soil Zn fractions among the treatments at the different periods were evaluated by one-way ANOVAs. The relative amount of ineffective Zn (*Zn*_*RI*_) was tested with one-way ANOVA to explore the effectiveness of straw application. All the ANOVAs were conducted by using the procedure of general linear model and the differences between means were compared by the least significant difference (*LSD*) method at the 5% level. In addition, to predict the ageing process of exogenous Zn with time, the linear regressions (Eq (5)) between the relative amount of ineffective Zn (*Zn*_*RI*_) and square root of time were calculated both in the Zn and Straw+Zn treatments. The relationship between the various Zn fractions and DTPA-Zn were assessed by a path analysis. Furthermore, the climatological data of air temperature and rainfall during the incubation period was determined in the experimental farm.

## Results

### Soil available Zn (DTPA-Zn) concentration

The addition of water-soluble Zn to the soil improved the available Zn concentrations significantly, but the effects of straw application were not significant except for the 60 days (Table 1, *P*<0.05). On average, a 2% increase in the mean DTPA-Zn concentration only was recorded for the straw application. Compared with the no Zn treatment, however, the marked increases of DTPA-Zn ranging from 3.9 to 5.9 folds (mean 4.9) were recorded for the Zn amendments and this increase effect decreased with the passage of time (Table 2). Weak effects of interaction between straw and Zn application on the DTPA-Zn concentrations were recorded except for the 195 days (Table 1, *P*<0.05). Furthermore, the reduction in DTPA-Zn concentration after exogenous Zn addition occurred from 120 to 165 days and from 180 to 210 days. However, the values of soil DTPA-Zn had a mild fluctuation with time in the absence of Zn treatments (Table 2). For example, in the control treatment, the values ranged from 0.65 to 0.86 mg kg^-1^over the whole periods and the variation coefficient (CV) was only 8%.

**Table 1:** Summary of two-way ANOVAs for the effects of straw, Zn application and their interaction on soil DTPA-Zn, pH, and labile organic carbon (LOC) at the various incubation periods.

Indicators	Source	df	*F* probability						
			60	120	150	165	180	195	210
**DTPA-Zn**	**Straw (A)**	1	0.048	0.962	0.310	0.780	0.261	0.186	0.718
	**Zn (B)**	1	<0.001**	<0.001**	<0.001**	<0.001**	<0.001**	<0.001**	<0.001**
	**A × B**	1	0.168	0.411	0.098	0.352	0.953	0.024*	0.294
**pH**	**Straw (A)**	1	0.062	0.066	0.416	0.321	0.487	0.563	0.084
	**Zn (B)**	1	0.436	0.603	0.075	0.432	0.051	0.936	0.042*
	**A × B**	1	0.057	0.328	0.057	0.921	0.938	0.529	0.142
**LOC**	**Straw (A)**	1	0.005**	0.083	<0.001**	<0.001**	<0.001**	0.233	0.041*
	**Zn (B)**	1	0.811	0.055	0.124	0.101	0.413	0.412	0.008**
	**A × B**	1	0.759	0.021*	<0.001**	<0.001**	<0.001**	0.642	<0.001**

Note

* means significant difference at *P*<0.05 for *F*-test

** means significant difference at *P*<0.01 for *F*-test

**Table 2:** DTPA-Zn concentration (mean ± SE, *n* = 3) of soils with the addition of straw and Zn at the various incubation periods.

Treatment		DTPA-Zn concentration(mg kg^-1^)						
		60	120	150	165	180	195	210
**0**	**0**	0.75 ± 0.03	0.77 ± 0.07	0.72 ± 0.08	0.65 ± 0.05	0.76 ± 0.05	0.75 ± 0.04	0.86 ± 0.01
	**Straw**	0.81 ± 0.08	0.69 ± 0.01	0.62 ± 0.04	0.73 ± 0.02	0.92 ± 0.06	0.98 ± 0.05	0.99 ± 0.02
**Zn**	**0**	4.65 ± 0.05	5.06 ± 0.15	4.57 ± 0.21	4.23 ± 0.19	4.72 ± 0.17	4.66 ± 0.05	4.60 ± 0.15
	**Straw**	4.87 ± 0.05	5.15 ± 0.11	4.43 ± 0.33	4.09 ±0.10	4.96 ± 0.20	4.77 ± 0.05	4.54 ± 0.08
***LSD***_**0.05**_ **of A or B**^a^		0.12	0.24	0.27	0.25	0.31	0.11	0.20
***LSD***_**0.05**_ **of A×B**^b^		0.18	0.33	0.39	0.36	0.45	0.15	0.28

Note:

^a^The value of LSD (least significant difference) for main effects of Zn (A) or straw (B) at 5%.

^b^The values of LSD for interaction between Zn (A) and straw (B) at 5%.

### Soil pH and labile organic carbon (LOC) concentration

Regardless the incubation period, the values of soil pH ranged from 7.83 to 8.23 (mean 7.98) among the treatments (Table 3). The marked influence of straw application on soil pH was only observed on days of 210. However, Zn application as well as the interaction between straw and Zn didn't affect the pH within the 210-day incubation (Table 1, *P*<0.05). For the LOC concentration, the significant increases after the addition of straw occurred in the days of 60, 150, 165, and 210. On average within the 210-day incubation, the LOC concentration increased by 5.4% both in the straw added treatments compared with the control treatment. However, response of soil LOC to Zn application was very weak at any incubation time except the days of 180 with a negative effect and 210 with a positive effect (Tables 1 and 3). Furthermore, the interaction between straw and Zn markedly affected on the LOC concentration at any time (except for the days 60 and 195) (Table 1, *P*<0.05).

**Table 3:** Soil pH and labile organic carbon(LOC) concentration affected by straw (A) and Zn application (B) at the various incubation periods.

Treatment		60	120	150	165	180	195	210
**pH**								
**0**	**0**	7.88 ± 0.03^a^	7.92 ± 0.03	7.83 ± 0.01	7.90 ±0.04	8.00 ± 0.02	8.09 ± 0.03	8.23 ± 0.07
	**Straw**	7.91 ± 0.01	7.94 ± 0.01	8.05 ± 0.02	7.95 ±0.05	7.86 ± 0.01	8.05 ± 0.05	8.10 ± 0.09
**Zn**	**0**	8.04 ± 0.03	7.88 ± 0.06	7.93 ± 0.02	7.97 ± 0.11	7.98 ± 0.02	8.08 ± 0.05	8.14 ± 0.10
	**Straw**	7.96 ± 0.03	7.83 ± 0.01	7.89 ± 0.06	8.04 ±0.07	7.84 ± 0.05	8.12 ± 0.08	8.00 ± 0.08
**LSD**_**0.05**_ **of A or B**^b^		0.08	0.10	0.08	0.07	0.09	0.06	0.13
**LSD**_**0.05**_ **of A×B**^c^		0.17	0.15	0.15	0.09	0.17	0.11	0.33
**LOC (g kg**^**-1**^**)**								
**0**	**0**	1.53 ± 0.01	1.55 ± 0.03	1.73 ± 0.11	1.80 ± 0.03	2.01 ± 0.06	1.74 ± 0.32	1.60 ± 0.00
	**Straw**	1.65 ± 0.02	1.58 ± 0.03	2.06 ± 0.05	1.98 ± 0.05	2.12 ± 0.11	1.56 ± 0.06	1.66 ± 0.02
**Zn**	**0**	1.53 ± 0.06	1.65 ± 0.01	1.92 ± 0.06	1.65 ± 0.24	1.86 ± 0.16	1.53 ± 0.04	1.71 ± 0.01
	**Straw**	1.61 ± 0.07	1.78 ± 0.03	2.02 ± 0.03	2.07 ±0.03	1.63 ± 0.12	1.66 ± 0.02	1.83 ± 0.04
**LSD**_**0.05**_ **of A or B**		0.09	0.06	0.13	0.10	0.11	0.08	0.06
**LSD**_**0.05**_ **of A×B**		0.16	0.11	0.20	0.16	0.18	0.20	0.11

Note:

^a^ Values are means ± SE, n = 3

^b^ The value of LSD (least significant difference) forma in effects of Zn (A) or straw (B) at 5%.

^c^The values of LSD (least significant difference) for interaction between Zn (A) and straw (B) at 5%.

### Relative amount of ineffective Zn (*Zn*_*RI*_), apparent diffusion coefficient (*D*_*a*_), and ageing rate of Zn fertilizer added to soil

**Fig 1:**
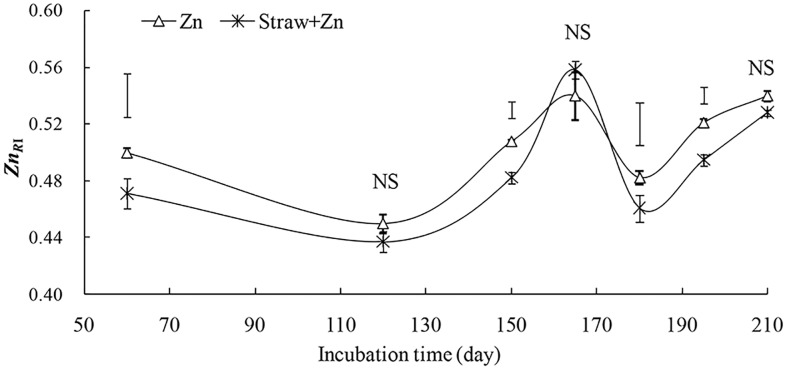
The relative amount of ineffective Zn (*Zn*_*RI*_) in soils with the water-soluble Zn application (Zn) alone and along with straw (Straw+Zn) at the various incubation periods. The bars on the curves are standard errors (*n* = 3) and the bars above the curves represent the least significant difference (*LSD*) at 5%.

According to the results of Table 2, soil DTPA-Zn had a small variation (CV = 8%) with the time passage in the control treatment, implying that soil endogenous DTPA-Zn concentration scarcely changed with time. We consequently considered the average value of 0.79 mg kg^-1^ to be the endogenously initial DTPA-Zn (equivalent to *Zn*_*soil*_ in the Eq (3)). Accordingly, the relative amounts of ineffective Zn (*Zn*_*RI*_) were calculated both in the Zn and Straw+Zn treatments and the values ranged from 0.437 to 0.539. One-way ANOVAs indicated that straw application significantly reduced *Zn*_*RI*_ after60, 150, 180, and 195 days (Fig 1, *P*<0.05). With the presence of exogenous Zn, the mean *Zn*_*RI*_ was 0.505 for the no straw treatment and 0.490 for the straw treatment. Furthermore, the increase in *Zn*_*RI*_ occurred from 120 to 165 days and from 180 to 210 days for both treatments (Table 4). The relation between *Zn*_*RI*_ and square root of time were not expressed well by the diffusion model for both Zn and Straw+Zn treatments over the whole time, but only at the two specific incubation periods of 120–165 and 180–210 (Table 4). When exogenous Zn was added, straw application reduced constant *A* which represents the initial sorption of Zn in soil (Table 4). At the day of 120–165, the initial sorption of Zn was17.5 μg Zn kg^−1^ soil for the Zn treatments but 6.3 μg Zn kg^-1^ soil for the Straw+Zn treatment. At the day of 180–210, the initial sorption of Zn was26.6 μg Zn kg^−1^ soil and 10.5 μg Zn kg^−1^ soil, respectively. For the values of *D*_*a*_, it decreased with increasing time for both treatments. Especially for the Zn treatment, this reduction was up to 52%. Additionally, Zn addition plus straw increased the *D*_*a*_ by 66% for 120–165 days and 232% for 180–210 days compared with the Zn addition alone. The values of ageing rate for the Straw+Zn treatment were greater than those for the Zn treatment at any incubation time (Table 5). For example, the ageing rate was 15.7 μg Zn day^-1^ (78 μg Zn kg^-1^ soil day^-1^) after the 120-day incubation and 8.21 μg Zn day^-1^ (41 μg Zn kg^-1^ soil day^−1^) after the 210-day incubation for the Zn treatment, whereas it was 20.1 μg Zn day^-1^ (101 μg Zn kg^-1^ soil day^-1^) after the 120-day incubation and 14.9 μg Zn day^-1^ (75 μg Zn kg^-1^ soil day^-1^) after the 210-day incubation with the Straw+Zn treatment.

**Table 4:** The parameters of diffusion equation in soils with the water-soluble Zn application (Zn) and along with straw (Straw+Zn) at the periods of 120–165 and 180–210 days.

Incubation periods (day)	Treatment	Parameters for diffusion equation				
		*A*	6 $\sqrt{D_{a}/\pi }$	*D*_*a*_ (day^-1^)	*r*	*P* value
**120–165**	**Zn**	0.0025	0.049	2.09 × 10^−4^	0.994	0.003
	**Straw+Zn**	0.0009	0.063	3.46 × 10^−4^	0.955	0.004
**180–210**	**Zn**	0.0038	0.034	1.01 × 10^−4^	0.975	0.023
	**Straw+Zn**	0.0015	0.062	3.35 × 10^−4^	0.989	0.008

**Table 5:** Ageing rate of water-soluble Zn in soils with the Zn application (Zn) and along with straw (Straw+Zn).

Treatment	Ageing rate (μg Zn day^-1^)						
	120	150	165	180	195	210	Mean
**Zn**	15.7	14.0	13.4	8.87	8.52	8.21	11.4
**Straw+Zn**	20.1	20.5	19.5	16.2	15.5	14.9	17.8

### Zn fractions

**Fig 2:**
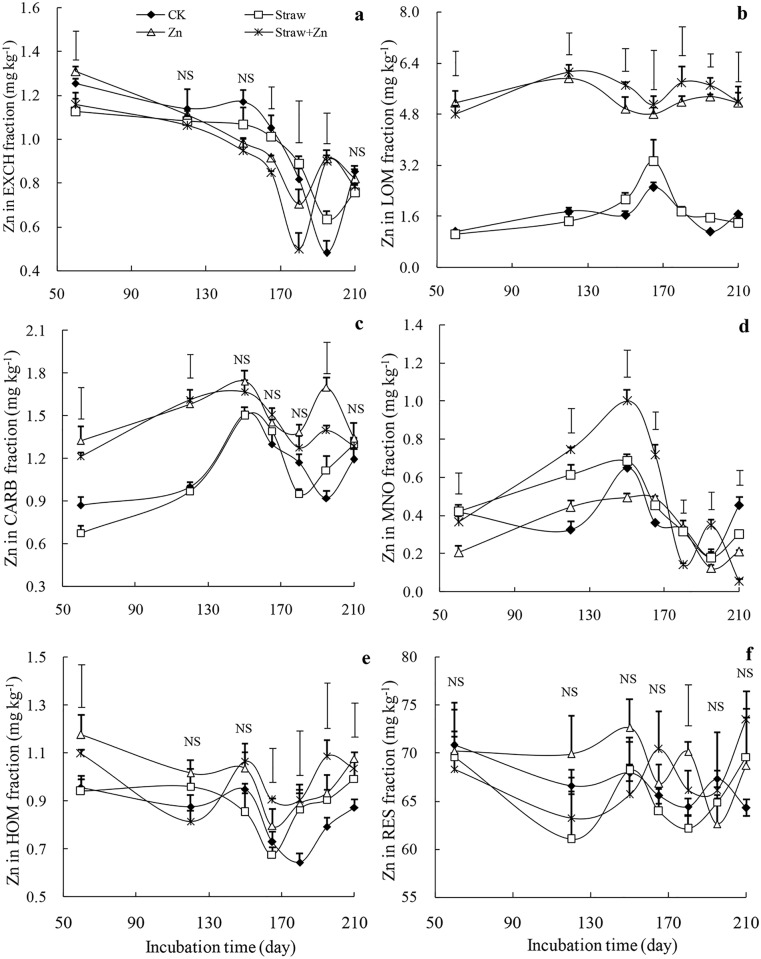
Zn concentration among the fractions in soils with exogenous Zn and straw applications. **Soil total Zn was separated into the Zn in exchangeable (EXCH, a), organic matter with low bonding forces (LOM, b), carbonate (CARB, c), manganese oxide (MNO, d), organic matter with high bonding forces (HOM, e), and residual (RES, f) fractions by a sequential extraction procedure**. The bars on the curves are standard errors (*n* = 3) and the bars above the curves represent the least significant difference (LSD) at 5% and "NS" means no significant difference.

Fig 2 showed the concentration of different Zn fractions in soil with the addition of straw and Zn. There were considerable variations of soil Zn in EXCH fraction among the treatments after 60, 165, 180 and 195 days. Compared with the control treatment, the EXCH fraction concentration decreased both for the Zn added treatments after 165 and 180 days, but increased after 195 days. During the whole incubation period, except for 210-day, its concentration decreased with the increase of time. Compared with 60 days, the largest decrease was 54% for the absence of Zn treatments within 195 days and 51% for the presence of Zn treatments within 180 days. Response of Zn in LOM fraction was strong to Zn application, but was weak to straw amendment (Fig 2, *P*<0.05). Regardless of incubation time, mean LOM-Zn fraction increased by 208% for the Zn application treatment, but by only 6% for the straw application treatment. Although the Zn in LOM fraction for the Straw+Zn treatment were greater than those for the Zn treatment, there were no differences between them for any of the incubation periods. In addition, the values of LOM fraction fluctuated with the increasing time of incubation; the largest concentration was measured after 165 days for the no Zn treatments and after 120 days for the Zn application treatments. Exogenous Zn affected the Zn bound to CARB fraction after 60, 120 and 195 days only. Regardless of treatments, the CARB fraction first increased and then decreased with time; the largest value occurred after 150 days in all treatments. Additionally, regardless of time, both straw and Zn applications increased the fraction of Zn bound to MNO before day 165, after which the increase became weak. The Zn in MNO fraction for all treatments increased first and then decreased with time. Compared with the control, Zn in HOM fraction increased for the Zn plus straw treatment at the any periods except for 120-and 150-day. The Zn in RES fraction and total Zn (data no shown) were not affected by straw and Zn applications over the whole time.

## Discussion

### Effect of straw amendment on soil Zn availability

The response of soil Zn availability to exogenous straw alone was very weak within 120-day incubation, which differed markedly from its response to other exogenous organic materials [25]. This difference might be related to the inherent Zn concentration of organic amendments. In general, the organic materials from farmyard manure or municipal solid waste has an inherently larger Zn concentration and consequently released abundant free Zn into soil through its decomposition, which resulted in an increase in soil Zn availability [9]. Although the straw application (27 Mg ha^-1^) of this study was1.5 times of the currently conventional ones(18 Mg ha^-1^), the used straw had an low Zn concentration (6.7 mg kg^-1^) and large C:Zn ratio (>65000). Free Zn released from straw might be overshadowed by Zn fixation by microbes for growth [26, 27]. Furthermore, the soil chemical and physical constraints, such as high pH, carbonate, and clay concentration, probably and adversely affected the response of straw amendment to Zn availability. In the present research, soil pH held a high level (~8) over the 210 days and was unaffected by adding straw alone (Table 3), due to the high pH buffer which derived from the high carbonate content in calcareous soil. The availability of trace elements were greatly controlled by the high soil pH because the increased pH enhanced soil negative charge and accordingly the adsorptive capacity of the soil solid surfaces, resulting in the formation of precipitation in carbonate and chemisorptions on clay minerals with more Zn [28]. A number of studies have shown that high soil carbonate and clay often precipitated or adsorbed Zn from soil solution, thus reducing Zn availability [29]. An increase in pH in alkaline soil can double the strength of bonding of Zn to calcite and soil minerals [30]. Although straw amendment alone increased the LOC concentration and consequently slightly enhanced Zn concentration in LOM fraction, the soil Zn concentration in CARB, LOM, and RES fractions were not decreased owing to high adsorption affinity of carbonate and clay for Zn at the high pH. Similar results were reported by Baldwin and Shelton [31], who found that exogenous organic matter did not affect the availability of trace elements in soil types with a strong affinity for microelements (i.e. soil with a large proportion of clay and carbonate). On the contrary, the increased crop yield resulting from the addition of straw further intensify the depletion and deficiency of soil Zn because of the lack of Zn fertilizer applied [32]. Consequently, under the current straw return system, soil amendments to modify soil properties such as pH, carbonate, and clay might improve the availability of Zn in calcareous arable soil. For example, sulphur and gypsum increased the availability and mobility of Zn in alkaline and calcareous soil through decreasing soil pH [3, 33].

### Effect of soluble Zn on soil Zn availability and fractions

Water-soluble Zn fertilizer alone increased the DTPA-Zn concentration (>4 mg DTPA-Znkg^-1^) in calcareous soil. When fractionation was used, it was found that the concentration of Zn in every fraction increased with Zn addition, but the availability of Zn in each fraction was different (Fig 1). Numerous studies indicated that when continuous extraction procedure was used, the availability of Zn fractions decreased with the increased steps [7]. The first fraction of Zn in EXCH is water-soluble and exchangeable and the second fraction contained Zn associated with both humic and fulvic fractions, and those two fractions were considered to be readily available [8, 10]. The fractionation showed that the Zn in EXCH fraction continuously decreased with time and no relation occurred with DTPA-Zn by path analysis (Fig 2, Table 6). However, Zn fraction in LOM for the Zn treatment was about 4.5-fold greater than that for the control treatment (Table 2). Zn fractions in LOM was significantly and positively correlated with DTPA-Zn concentration (*r* = 0.964, *P*<0.01) and its direct path coefficient for DTPA-Zn was up to 0.969 (Table 6). Furthermore, the indirect path coefficients indicated that other fractions could affect DTPA-Zn through Zn fractions in LOM and their indirect path coefficients for DTPA-Zn had the order: CARB > TOM > MNO > RES > EXCH. For Zn fraction in CARB, it was positively related to DTPA-Zn (*r* = 0.603, *P*<0.01) but its direct path coefficients (-0.134) was negative (Table 6). These revealed that the positively indirect effect of Zn in CARB on DTPA-Zn through Zn fraction in LOM was predominant rather than the negative direct effect. Additionally, when exogenous Zn was freshly added, the increase of Zn fractions in CARB, MNO, TOM, and RES were very limited (Fig 2). These results implied that native organic matter (especially humic substance) played a key role in the availability of exogenous Zn in calcareous soil and had a more dominant presence in Zn reaction than soil other constituents such as carbonate and minerals, although the tested soil always had a high pH, carbonate and clay content. This was in agreement with the study of Donisa et al. [34] who reported that the availability of soil native Zn was strongly controlled by Zn complexes with humic substances.

**Table 6:** Path coefficients and correlation coefficient of soil Zn fractions in exchangeable (EXCH), organic matter with low bonding forces (LOM), carbonate (CARB), manganese oxide (MNO), organic matter with high bonding forces (HOM), and residual (RES) for DTPA-extractable-Zn. The coefficient of determination and the residual path coefficient were 0.962 and 0.195, respectively.

Fractions	Correlation coefficient	Direct path coefficient	Indirect path coefficient					
			→EXCH	→LOM	→CARB	→MNO	→TOM	→RES
**EXCH**	-0.091	-0.097	**−**	-0.049	-0.003	0.017	0.038	0.003
**LOM**	0.964**	0.969	0.005	**−**	-0.095	0.011	0.062	0.008
**CARB**	0.603**	-0.134	-0.002	0.689	**−**	0.022	0.023	0.004
**MNO**	0.209	0.041	-0.042	0.278	-0.073	**−**	0.012	-0.001
**TOM**	0.522	0.156	-0.024	0.388	-0.020	0.003	**−**	0.019
**RES**	0.287	0.041	-0.008	0.198	-0.013	-0.001	0.072	**−**

Note

* means significant difference at *P*<0.05

** means significant difference at *P*<0.01.

**Fig 3:**
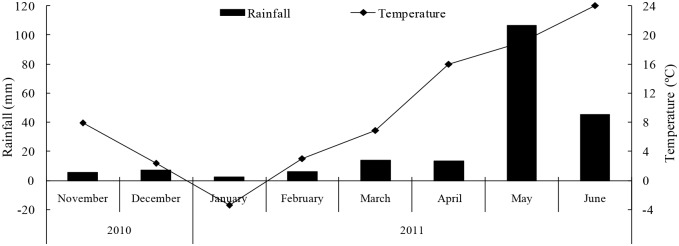
Monthly mean air temperature and accumulated rainfall during the incubation period.

It was notable that Zn fractions in CARB, MNO, TOM and RES dramatically fluctuated with the continuously decreased Zn fraction in EXCH within 210days incubation (Fig 2). This greatly countered the results of Ma and Uren [4, 24], who found the Zn fractions in easily reducible Mn, carbonate, stable organic matter, and residue continuously increased with the reduction in Zn in water soluble plus exchangeable and EDTA extractable fractions in the green house experiments where the soluble Zn was added into calcareous soils. We attributed this to the difference of environmental conditions. The present study was conducted in the field with a great variation of soil temperature and moisture that resulted from the interaction of air temperature and rainfall, consequently altering the response of exogenous Zn availability to time. Zn deficiency is more prevalent when soils are cold and dry [35, 36]. During the whole incubation time, the monthly mean temperature markedly ranged from -3.4 to 24°C and monthly accumulated rainfall from 5.3 to 106 mm (Fig 3). Within the first 165 days incubation, the continuously increased Zn forms in CARB and MNO might result from the lower soil temperature and moisture due to the lower air temperature and rainfall. However, Zn in EXCH fraction was continuously decreased within the 210–day, indicating that the transformation of water soluble and exchangeable Zn into less readily available forms was only time-dependant and wasn't influenced by environmental conditions. Accordingly, the contribution of Zn in EXCH fraction to Zn bioavailability was very limited owing to its ephemerality. For Zn fraction in LOM, its concentration with adding exogenous Zn held a high level (>4.5 mg kg^-1^) and fluctuated mildly with the increased time, which meant Zn fraction in LOM was long-lived in calcareous soil, although environment conditions changed dramatically.

Consequently, the addition of organic amendments might further increase the response of Zn availability to exogenous Zn through enhancing the Zn fraction in LOM in calcareous soil.

### Effect of straw amendment on ageing of exogenous water soluble Zn

Both water-soluble Zn alone and together with straw added into the soil increased the available Zn concentration, but their relative amount of ineffective Zn(*Zn*_*RI*_) was up to ~50% of the total added fertilizer-Zn. Within the 210-day,thediffusion model(Eq (5))did not express the relations between the values of *Zn*_*RI*_ and time both for Zn added treatments. This markedly differed from the results of an indoor incubation study conducted by Ma and Uren [24], who found that the *Zn*_*RI*_ in calcareous soil increased continuously with time within 2-yearincubation and the relationship was aptly described by the diffusion model when soluble Zn was added. Additionally, Jalali and Khanlari [7] also found the reduction of the available Zn forms over 28 days laboratory incubation fitted well the diffusion equation in calcareous soil mixed with soluble Zn. However, for the Zn and Straw+Zn treatments, a significant reduction in *Zn*_*RI*_ along with the increase of DTPA-Zn concentration occurred at the days of 60–120 and 165–180 in the present study (Fig 1), which might be related to the dramatic change of the temperature and rainfall which affected the ageing process of exogenous Zn [37]. Wang et al [38] found soil freezing and thawing cycle with the increased temperature significantly enhanced the Zn availability. The DTPA-Zn concentration enhanced with the increased soil moisture [36]. During the days of 60–120, the increase of Zn fractions in LOM and the reduction in Zn fractions in RES and TOM resulted in a significant reduction in *Zn*_*RI*_, resulting from the continuously increased temperature and rainfall from January to March in 2011 (Figs 2 and 3). The reduction in *Zn*_*RI*_ for the days of 165–180 might be attributed to the sudden and high rainfall (97 mm) in the first half of May 2011 (data no shown). On the contrary, during the incubation days of 120–165 and 185–210, the diffusion model (Eq (5)) expressed well the relations between the values of *Zn*_*RI*_ and time both for Zn added treatments (Fig 2). This results suggest that diffusion of available Zn is the rate-limiting step for increasing Zn availability in calcareous soil within the 120–165 and 185–210 days only when the air temperature and rainfall had a mild variation. Accordingly, the diffusion equation is effective to evaluate the process of exogenous Zn ageing in calcareous soil, but should be cautiously adopted in the sharply changeable environment conditions such as temperature and rainfall with time.Adding Zn together with straw increased the soil initial sorption of Zn (see the constant *A* in Table 4) and Zn apparent diffusion coefficient compared with adding soluble Zn alone. This result implied that the higher initial sorption of Zn was in soil, the higher apparent diffusion rate became. In our study, although straw amendment significantly increased the LOC concentration, it slightly enhanced the Zn fractions in LOM when exogenous Zn was added to soil (Table 3, Fig 2). This results indicated that the LOC from exogenous organic material had a little effect on Zn fraction in LOM. The ageing of exogenous water-soluble Zn was generally characterised by two stages: a initiallyrapid Zn sorption, which represents true Zn adsorption on mineral surface, followed by a slowly continuing Zn sorption reactions due to the diffusive penetration or chemisorptions of surface-adsorbed Zn into soil constituents and the precipitation of Zn compounds. Jalali and Khanlari [7] found the exogenous soluble Zn added to calcareous soil was homogenized and distributed into carbonate, Mn and Fe oxide, stable organic matter, and residue fractions within 7-day laboratory incubation, and then these fractions mildly changed with time. In our study because the low air temperature and rainfall greatly restricted straw decomposition, the hysteresis for the increase of LOC adversely affected the response of Zn fraction at the first stage [39]. Furthermore, the increase of Zn fraction in LOM was very limited with increasing the concentration of LOC due to the large affinity of CARB, MNO, RES to Zn at the second stage. Despite all these above mentioned, the slight increase in Zn fraction in LOM due to the soluble organic Zn complexes reduced the initial sorption of Zn and increased in the mobility of Zn in the Straw+Zn treatment to some extent. Furthermore, compared with the Zn treatment the 50% increase in mean ageing rate of Zn fertilizer in the Straw+Zn treatment (Table 5) might have resulted from increased Zn mobilization. Rapid ageing of added soluble Zn usually occurs with strong available Zn movement in soil with a large adsorption and fixation capacity such as calcareous soil.

## Conclusions

Zn availability had a very weak response to short-term (210-day) straw incorporation alone. However, water-soluble Zn alone improved the soil DTPA-Zn concentration through the increase of Zn fraction bound to LOM, which was unaffected by soil properties such as pH, carbonate and clay content, and environmental conditions. The values of *Zn*_*RI*_ indicated that about half of the Zn fertilizer was transformed into DTPA-unextractable-Zn fractions after 60–210 days incubation. The diffusion model (Eq (5)) didn't aptly evaluate the process of exogenous Zn ageing both in the Zn and Straw+Zn treatments over 210days, but fit well at the 120−165 and 180−210 days when the air temperature and rainfall had a mild variation. Adding soluble Zn together with straw increased the rate of Zn ageing in calcareous soil compared with adding soluble Zn alone, which may be attributed to its higher diffusion of available Zn. Consequently, combined the soluble Zn with straw returning is effective to enhance the Zn availability and mobility in calcareous soil, but considering the rapid ageing of exogenous Zn, further studies of plant bioavailability are recommended to confirm the results. Additionally, in non-calcareous soils, whether the diffusion equation is appropriate to evaluate the process of exogenous Zn ageing may need further study.
